# Deployment of a Free-Text Analytics Platform at a UK National Health Service Research Hospital: CogStack at University College London Hospitals

**DOI:** 10.2196/38122

**Published:** 2022-08-24

**Authors:** Kawsar Noor, Lukasz Roguski, Xi Bai, Alex Handy, Roman Klapaukh, Amos Folarin, Luis Romao, Joshua Matteson, Nathan Lea, Leilei Zhu, Folkert W Asselbergs, Wai Keong Wong, Anoop Shah, Richard JB Dobson

**Affiliations:** 1 University College London London United Kingdom; 2 Institute of Health Informatics University College London London United Kingdom; 3 National Institute for Health and Care Research Biomedical Research Centre University College London Hospitals National Health Service Foundation Trust London United Kingdom; 4 Health Data Research UK London University College London London United Kingdom; 5 National Institute for Health and Care Research Biomedical Research Centre South London and Maudsley National Health Service Foundation Trust King’s College London London United Kingdom; 6 Department of Biostatistics and Health Informatics Institute of Psychiatry, Psychology and Neuroscience King’s College London London United Kingdom; 7 Epic Systems Corporation London United Kingdom

**Keywords:** natural language processing, text mining, information retrieval, electronic health record system, clinical support

## Abstract

**Background:**

As more health care organizations transition to using electronic health record (EHR) systems, it is important for these organizations to maximize the secondary use of their data to support service improvement and clinical research. These organizations will find it challenging to have systems capable of harnessing the unstructured data fields in the record (clinical notes, letters, etc) and more practically have such systems interact with all of the hospital data systems (legacy and current).

**Objective:**

We describe the deployment of the EHR interfacing information extraction and retrieval platform CogStack at University College London Hospitals (UCLH).

**Methods:**

At UCLH, we have deployed the CogStack platform, an information retrieval platform with natural language processing capabilities. The platform addresses the problem of data ingestion and harmonization from multiple data sources using the Apache NiFi module for managing complex data flows. The platform also facilitates the extraction of structured data from free-text records through use of the MedCAT natural language processing library. Finally, data science tools are made available to support data scientists and the development of downstream applications dependent upon data ingested and analyzed by CogStack.

**Results:**

The platform has been deployed at the hospital, and in particular, it has facilitated a number of research and service evaluation projects. To date, we have processed over 30 million records, and the insights produced from CogStack have informed a number of clinical research use cases at the hospital.

**Conclusions:**

The CogStack platform can be configured to handle the data ingestion and harmonization challenges faced by a hospital. More importantly, the platform enables the hospital to unlock important clinical information from the unstructured portion of the record using natural language processing technology.

## Introduction

### Background

Over the past 20 years, we have seen an increased uptake of electronic health records (EHRs) within health care organizations, with much of this being attributable to national efforts in having health care organizations transition to using full EHR systems [1,2]. These EHRs represent a rich data asset, but there remains a challenge in the secondary use of the data for improving clinical care through activities, such as service improvement and clinical research. In many cases, EHRs have simply replicated the paper system that they replaced and have not taken full advantage of the opportunities presented in having the health records in this new electronic format. While functional systems to address these gaps are emerging, many of the tools and data analytic approaches used on EHR data are limited to structured data, such as coded diagnoses and numeric clinical measurements. However, the structured data only account for a small portion of the EHR data, as it is estimated that almost 80% of information records remain unstructured in the form of images, free-text records, and other such unstructured data formats [3]. In particular, the free-text records often contain important clinical information, such as patient diagnoses, that have not yet been recorded as structured data [4]. An additional difficulty is that a hospital’s record is typically distributed across numerous disconnected data systems, which presents a challenge in data harmonization.

Working with EHRs thus presents challenges firstly in harmonizing and accessing the hospitals entire record from both existing and legacy data systems and secondly having tools and techniques available to mine and extract data from within these records, especially the unstructured free text. Manual analysis of unstructured text is time-consuming, so there has been much interest in developing automated methods for extracting accurate structured information from the free-text records [5]. Interpreting free text is a major analytic challenge; clinical text is written in a variety of styles by numerous authors and may have misspellings, negations, and other linguistic features. There has been intense interest in developing natural language processing (NLP) techniques to interpret clinical text [6,7]. Early methods used a rule-based approach, but more modern algorithms incorporate machine learning techniques, enabling the algorithms to “learn” as more data are analyzed.

The CogStack platform [8] was developed to address these exact problems. The platform can be described as an information retrieval system designed to interface with a hospital’s EHR system. It was initially developed with an emphasis on ingestion and harmonization of records from multiple data systems within a health care organization. While certain off-the-shelf NLP tools were explored in the first iteration, they were added as a proof of concept to demonstrate that the platform could potentially be configured to interact with such tools.

In this paper, we discuss the experience of deploying CogStack at University College London Hospitals (UCLH) and highlight modifications to the platform that have improved its data harmonization and NLP capabilities. Our deployment of CogStack has focused on addressing the following 3 key issues that we feel are universal to all research driven health care organizations.

### Multiple Data Systems

The EHRs of an organization will typically be distributed across a number of different vendor systems, posing a challenge for the use of this information for clinical care and research. It is not uncommon for an organization to have to maintain oversight over a myriad of data systems and vendors due to the fact that different clinical specialties will have different requirements of how data needs to be stored and managed. The resulting heterogeneity in data means that it is challenging for the organization to find a common data model or even process through which the organization’s entire record can be harmonized. Methods and systems through which data are stored, collected, and retrieved have been improving in order to tackle this challenge. Most notably, many National Health Service (NHS) trusts have opted to transition to using full-scale EHR systems (eg, Epic), each of which typically enforce their own data models. Some systems, such as Epic, go further in providing additional systems that allow data from third-party data and legacy systems to be integrated with data collected via their own systems (Epic Clarity/Caboodle). Messaging standards (eg, HL7 Fast Healthcare Interoperability Resources [FHIR] [9]), standardized terminologies (eg, Systematized Nomenclature of Medicine -- Clinical Terms [SNOMED CT]), and standardized clinical information models (eg, openEHR archetypes [10]) aim to improve interoperability between systems, but much more work is needed in this area. In order to maximize the benefit of patient data, it is essential that clinicians and researchers can access data in a way that is flexible, easily adaptable, and independent of the organization’s choice of current and previous EHR systems.

### Multiple Data Formats

A patient’s record may be distributed across both scanned documents (PDFs) and text documents (.doc files), and data may be stored in relational databases. Legacy documents, for example, will likely be stored as files and attachments, whereas data that have been generated using a modern EHR system will likely be stored in a more structured way, possibly in a relational database. An information retrieval system would thus need to be able to ingest and interact with records from all the various data formats used by the organization. The CogStack platform provides functionality for document processing, including PDF to text conversion, or optical character recognition that may be needed prior to analysis of the text itself.

### Unstructured Text

A final issue is that data within the EHR systems are recorded in both structured and unstructured fields. Some information is inherently unstructured in nature and needs to be recorded as free text (eg, patient stories), but even where structured fields are available, clinicians may not use them and enter the information in free text instead. For example, a recent audit in our trust found that patients admitted with suspected or confirmed COVID-19 had only 62.3% of their key diagnoses and comorbidities recorded in the structured problem list [4]. In order to support use of clinical data at scale and for multiple stakeholders, a successful information retrieval system should provide mechanisms through which the clinical information within the unstructured free-text notes can be made available. The CogStack platform provides a convenient user interface for searching free text, invoking information extraction algorithms, and presenting the results in a way that is easy to visualize and harness for downstream research or for reintegration as structured data back into the EHR.

There has been a great deal of interest in integrating NLP systems with EHRs to tackle the problem of unlocking value from unstructured data [11]. A number of commercial vendors have proposed NLP analysis as a service, where the vendor supplies NLP models that are used to process unstructured data [12-14]. In general, to our understanding, the NLP engines used by these vendors are trained using nontrust data and are generally not easily fine-tuned. In contrast, CogStack is a fully open-source platform, and the underlying NLP technology is tuned using the hospital’s data and deployed on hospital infrastructure. Furthermore, the intellectual property for the NLP engines is not owned by the vendor and instead is proprietary to the hospital.

In the rest of the paper, we describe the deployment of CogStack at UCLH and demonstrate how it has been configured to handle commonly seen use cases within the hospital. In the Results section, we demonstrate that it has been or is being currently used to support several service evaluation and research projects within the hospital.

## Methods

### Overview

**Figure 1:**
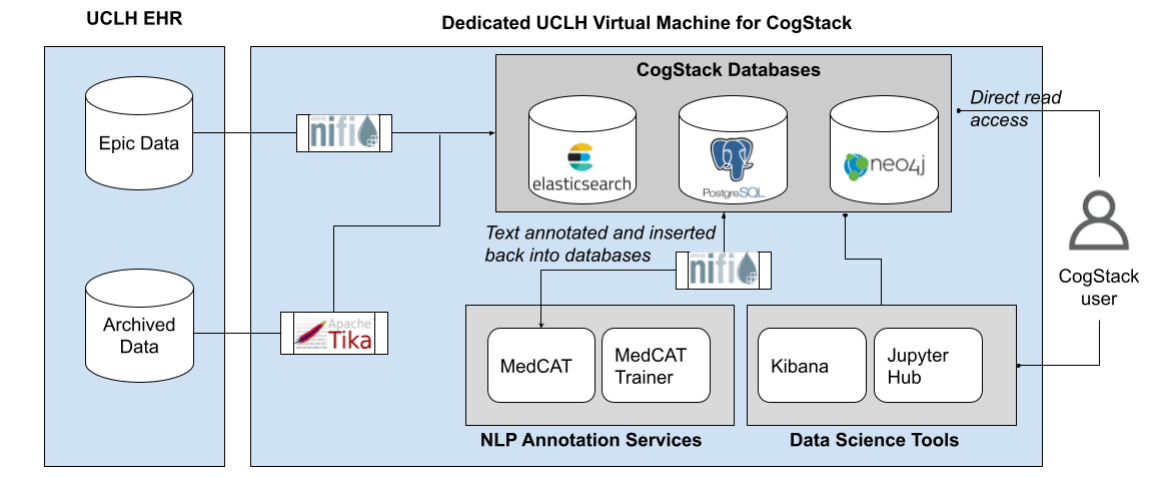
An overview of the CogStack platform as deployed at University College London Hospitals (UCLH). EHR: electronic health record; NLP: natural language processing.

In this section, we describe the various components of the CogStack platform [15] and describe how the platform has been deployed and configured at UCLH. Figure 1 depicts the various components and how they have been configured at UCLH. Broadly speaking, the platform provides 3 categories of functionality, namely, the ability to read data from the hospital’s EHR system, to store data, and to interact with the data programmatically on various NLP tools and interfaces.

### Infrastructure

The CogStack platform deployed at UCLH builds upon the previous version [8] that has been deployed at multiple hospitals, including South London and Maudsley Hospital, Guy’s and St Thomas’ Hospital, and King’s College Hospital. In particular, the latest version provides 2 key updates. The first is related to the improvements in the platform’s NLP capabilities, and the second relates to the use of Apache NiFi to manage the various data flows within the platform.

The first update is the use of the MedCAT NLP toolkit to provide clinical concept detection capabilities. The MedCAT tool is used to detect and extract clinical information from the free-text records (diagnosis, procedures, etc). The second update is the use of Apache NiFi for managing data flows within the platform. This was added based on lessons learnt from the previous iteration of CogStack, where the platform required the development of a number of custom extract, transform, and load (ETL) scripts for managing the ingestion of data from the live record and legacy systems. This approach however does not scale well in practice, and it can quickly become burdensome for developers to manage the various ETL scripts when the number of data flows increases. Observing these difficulties, the Apache NiFi module was added to the CogStack platform. Apache NiFi is a visual interface for managing complex data flows between different data systems. Data flows in Apache NiFi are depicted as directed graphs and provide useful visual feedback for system administrators, such as the status of a particular data flow and the number of documents processed. Most importantly, Apache NiFi is compatible with various data systems, which means that administrators are capable of writing the various ETL components in practically whatever programming language they choose. In addition, all of the data flows are accessible within a single interface, and this makes maintaining oversight of all of the data flows considerably easier than having to monitor multiple custom ETL scripts. UCLH has developed a number of NiFi workflows that are designed to work with the UCLH data warehouse as well as legacy data systems. These NiFi components conduct the various extractions and data transformations necessary for downstream CogStack services. We discuss these various data flows in the next section.

### Data Security and Governance

Use of unstructured EHR data for clinical research is challenging because of confidentiality concerns, leading to difficulty in obtaining ethics and information governance approvals for accessing such data. The CogStack approach is to embed text analytic capabilities and research staff within NHS Trusts, allowing sensitive text to be analyzed in situ.

Although data are routinely ingested into the CogStack platform, researchers wishing to use the data still need to undergo an approval process before accessing the data or making use of machine learning models trained on patient data for their research. UCLH has in place a system called Data Explorer [16] through which researchers can apply for access to use clinical data. If researchers require CogStack, an application needs to be submitted through the Data Explorer system and approved, and the appropriate data protection impact assessments (DPIAs) need to be completed. Each DPIA is assessed and approved by UCLH’s information governance lead before the user is able to access the data on the CogStack platform, and eventually, the permission to process and analyze the data using CogStack-trained machine learning models is provided.

As data ingested into the CogStack platform involve patient-sensitive information, all UCLH CogStack services are hosted within a secure environment that is only accessible within the hospital network. CogStack has a number of virtual machines that have been provisioned to process the trust’s data. We have followed the best practice for software deployment and have designated these virtual machines for development, testing, and production.

In addition, we have in place processes to be able to remove patient-identifiable data from the free-text records before use for research. CogStack has a deidentification module that is used to prepare batches of data for specific users and can be deployed before or after ingestion into CogStack’s central standardized data lake. The module builds on the open-source Philter library developed by the University of California, San Francisco, which achieved over 99% recall on the benchmark I2B2 deidentification data set by using a combination of rule-based and statistical approaches [17]. In the following text, we detail the ingestion pipeline as well as how data are accessed and processed once ingested into the platform.

#### Data Ingestion

CogStack uses Apache NiFi for managing data flows from the hospital’s various data sources into CogStack’s databases. In Figure 1, data flows can be seen between the live EHR (Epic data) and the hospital’s archived data warehouse. Table 1 provides a summary of the number of documents ingested so far into the platform. Using Apache NiFi, we are able to define how the ETL processes are implemented for each data source. We are able to set up data flows that run periodically, as well as manage ingestions that only happen once. Below, we describe the various data sources from which we ingest data.

**Table 1:** Number of notes ingested and analyzed by CogStack.

Document type	Number of notes
Clinical notes	10,500,000
Imaging reports	2,000,000
Clinical letters	3,000,000
Archived records	16,000,000

#### Trust Data

In 2019, UCLH officially transitioned to using Epic [18] as its primary EHR system. Prior to this, the trust had a number of data systems for each of its departments/clinics. The Epic system has in place a number of databases that capture integrated hospital data. Its data warehouse, Caboodle, has been extended to capture non-EHR and historic data records as well. UCLH has deployed the Epic Caboodle data warehouse for this purpose, and this is the primary database that the CogStack platform ingests data from.

As these data are stored as relational data, setting up data flows into CogStack requires only that CogStack understands the data schema of the target database. The data flows are then set up using Apache NiFi as a batch process. The batch process transforms the data into a format that is compatible with the various CogStack databases. Most clinical research projects requiring CogStack to date have been retrospective studies and have not required access to a live data feed. Consequently, the batch process runs on a daily basis, and this can be easily modified as needed through the NiFi interface.

#### Archived Data and Other Records

A number of records in the trust (such as those created prior to the transition to Epic) are not included in the Epic Caboodle data feed and require custom data flows to be set up. Records in the legacy systems are often stored as documents that have been scanned as images or as text documents (eg, .doc files, .pdf files, etc). In such cases, CogStack uses Apache Tika’s optical character recognition software to convert the contents of these documents into text that can then be saved into the platform’s various databases.

#### One-Off Ingestions

While CogStack’s primary focus is ingesting and processing data from the trust, there are occasionally requests to analyze nontrust data sets. Examples of this include allergy reports taken from the National Reporting and Learning System. In such cases, CogStack can accommodate these ad hoc requests via custom ingestion scripts using Apache NiFi.

#### Data Storage

As can be seen in Figure 1, the CogStack platform at UCLH saves its ingested data into 3 types of databases. This is to cater to the needs of the different types of users/downstream consumers of CogStack data. Once ingested, data can subsequently be accessed directly via a read-only user account or by using the set of data science tools that CogStack provides.

The first database provided is the ElasticSearch database, which is particularly useful for users and applications working with free-text data owing to its text-based indexing and querying capabilities. The second database is a PostGres database, which allows relational modeling of data and is more importantly widely compatible for many downstream users. Lastly, there has been recent work in ingesting data into a Ne04j database. This is to support the storage of graph-like data structures (eg, SNOMED ontology relations).

#### NLP Services

The core NLP functionality of the platform is provided by the MedCAT NLP toolkit [19]. The MedCAT toolkit is a named entity recognition and linking model that can identify clinical concepts in free text and link them to a predefined medical ontology (eg, SNOMED CT and UMLS). Currently, a UCLH-trained MedCAT model is deployed as a RESTful application programming interface (API) service and is scheduled via the Apache NiFi module to batch annotate new documents that have been inserted into the CogStack databases.

The underlying approach used by MedCAT is dependent on a neural network–based approach that learns latent representations (concept embeddings) of clinical concepts based on how they appear in free text. The underlying algorithm is a modified version of the word2vec algorithm, which learns numerical representations of a word based on the words that surround it.

Training MedCAT is done in 2 phases. The first phase is a self-supervised phase in which MedCAT employs a simple technique to preannotate a large corpus of clinical text. In this step, the algorithm identifies string matches for each concept synonym in the medical ontology being used (eg, searching for matches of “lung cancer” in each document). Once identified, the word2vec algorithm is used to learn embeddings for those identified entities within the documents. This process provides MedCAT with an initial representation for how the concepts are represented in free text.

In the second phase, the model is fine-tuned using human-provided annotations. In this case, the model is taught to predict the correct label as provided by the human annotator using the MedCAT trainer interface. Based on some previous studies [19], the number of annotations required for fine-tuning is small (500-600 annotated documents).

Collecting annotated data for training machine learning models is done through a custom annotation interface. A custom interface was chosen over off-the-shelf ones (eg, Doccano) as many of our annotation use cases require integrated tools for searching for clinical information.

MedCAT is trained using the MedCAT trainer interface [20]. The interface allows a user to load documents to be annotated by multiple annotators. The interface also provides an active learning mode that enables generated annotations to be used to retrain an existing MedCAT model in real time. The performance of the model can also be tracked in real time so the users can monitor performance change with additional annotations.

In addition to identifying clinical concepts in text, MedCAT provides a wrapper for training additional machine learning models for identifying important meta information for the extracted entities. Meta information of interest may include entity negated (eg, “patient does not have fever symptoms”), if an identified entity relates to the patient or to somebody else (the experiencer), or whether it is current or historic. In order to implement these models, MedCAT uses a sequence-based classifier (Bi-LSTMs) that takes the surrounding words of the identified terms and trains a classifier to predict if the meta label is assignable or not.

As mentioned earlier, at present, MedCAT is used to annotate documents that have been ingested into the platform. The annotations are saved in all 3 databases to ensure the end users have the ability to query whatever database they wish to use. The MedCAT models are trained using unsupervised learning based on records ingested into the platform. The model is occasionally fine-tuned when clinicians submit annotations via the MedCAT trainer interface. It is also useful to note that MedCAT models have been shown to generalize well across multiple hospital settings with only minimal fine-tuning required [19].

### Data Science Tools

The CogStack platform also provides data science tools for users to be able to interact with the platform’s data, as seen in Figure 1. Typically, users are either clinical researchers or data scientists, and the UCLH platform provides tools catering to both types of users.

**Figure 2:**
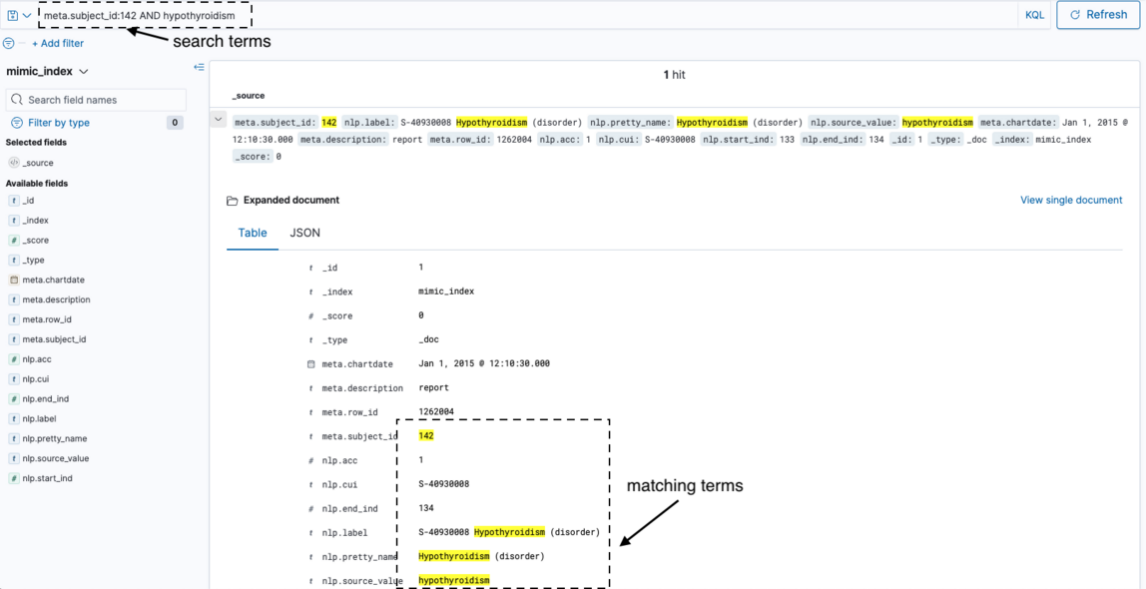
The Kibana interface being used to conduct keyword searches.

For use cases where querying via keywords and other easy-to-define features and regular expressions are enough, CogStack provides the Kibana interface (Figure 2). The Kibana interface provides a view of the data that have been ingested into the ElasticSearch index. Kibana provides a free-text search query interface in which the user can search across ingested documents using keywords and phrases. Compound queries can be created by using Boolean operators as well. In addition to its search functionality, Kibana provides some basic visualization tools that can be used to export basic charts and graphs from the data. Users of Kibana are given information via user manuals and an induction session on how to query their respective data sets using Kibana.

In many cases, however, users may desire more control over how they interact with the data. For example, certain users, particularly data scientists, will find aggregating and analyzing NLP annotations stored on the CogStack databases easier if done programmatically. In such cases, the CogStack platform provides a JupyterHub instance [21]. The JupyterHub provides the user the ability to interact with the record using various programming languages, including Python and R. User accounts on the JupyterHub instance are preloaded with a number of starter notebooks/scripts, which demonstrate how to connect the CogStack databases and how to interact with the NLP models.

## Results

### Overview

The CogStack platform has been used to facilitate several clinical research and service evaluation projects, which we describe below.

### Clinical Trial Recruitment

We used the CogStack platform in a retrospective simulation of patient recruitment in the LeoPARDS clinical trial [22], which studied a time-sensitive treatment for sepsis. We used NLP on free-text clinical notes from the intensive care unit at UCLH to identify mentions of infection and medical diagnoses relevant to inclusion and exclusion criteria for the trial [23]. We then applied a rule-based algorithm to identify eligible patients using a moving 1-hour time window, and compared patients identified by our approach with those actually screened and recruited for the trial.

Our method identified 376 patients, including all 34 patients with EHR data available who were actually recruited to LeoPARDS at the hospital. The sensitivity of CogStack for identifying patients screened manually was 90% (95% CI 85%-93%). Of the 203 patients identified by both manual screening and CogStack, the index date matched in 95 (46.8%) and CogStack was earlier in 94 (46.3%). We concluded that the CogStack platform with incorporated NLP could aid patient recruitment in a clinical trial, could identify some eligible patients earlier than manual screening, and could potentially improve trial recruitment by automatically identifying candidate patients if implemented in real time.

### NLP at the Point of Care

UCLH has recently been involved in a national program to develop an NLP system that can convert a clinician’s text into structured information in real time and extract information on diagnoses, medications, and allergies. The new NLP system will communicate with the “NoteReader” user interface component in Epic, which will allow clinicians to invoke the NLP system on their newly created clinical notes and generate structured information, which can be verified before it is committed to the record. The current workflow for clinicians involves writing the clinical note and then proceeding to manually input information on diagnoses, comorbidities, medications, and allergies into the appropriate structured fields.

The NLP system will use a trained MedCAT model, which will communicate with Epic NoteReader via a RESTful API. We have so far trained a MedCAT model on the entire UCLH record, which includes clinical notes, such as admission clerking and discharge summaries. Specific training tests included patients with COVID-19 [24] and patients with heart failure, and in each case, the model was trained to extract all diagnoses and symptoms, although for this project, the output will be filtered to include only extracted concepts that clinicians would find useful to include on the problem list.

### National Incident Reporting Database

We used CogStack as part of a detailed analysis of adverse reaction reports submitted to the National Reporting and Learning System. The work focused on identifying reasons for why patients had an allergic reaction to prescribed or administered medications. The CogStack platform was used to collect annotations and train a multiclass classification model using sentence embeddings to identify a number of themes and causes that may have been involved, directly or indirectly, in the patient’s adverse reaction.

The clinical collaborator, a consultant pharmacist, annotated a set of around 150 reports and labeled each report with one or more reasons for allergic reaction. A total of 20,788 incidents were extracted between January 01, 2012, and December 31, 2016. Six key themes were identified, including time (night and out of hours); documentation (source, completeness, and conflicts); knowledge (patient, medicine, and cross-sensitivity); external or system factors (guidelines, microbiology advice/results, and visual prompts); internal or individual factors (clinical condition, policy, procedure noncompliance, and considered decision-making); and medical/prescribing system (electronic or paper-based). A total of 170 allergy reports were annotated and used to train the model.

The macro-F1 was 0.62 across all subthemes. The model reported higher F1s for simpler themes, such as temporary staff (1.0) and microbiology advice (0.93), whereas for more complex themes, such as noncompliance to policy (0.45), the reported F1s were lower. This was because unlike the simpler themes, the complex themes could not be identified through keywords/phrases, and the number of training examples in the data set was too low for the model to be able to learn general semantic patterns for these themes.

### Improving the Clinical Referral Process for Neurology Clinics

Normal pressure hydrocephalus (NPH) is a condition that typically has a delayed diagnosis. CogStack has been used for a longitudinal study of symptoms in patients who attended the NPH clinic. The study allowed clinicians to build up a history of symptoms for each patient and understand better in what sequence symptoms typically occur before patients visit the clinic. The longer-term objective of the project is to use the analysis from the project to build alerting systems that can automatically suggest patients for the NPH clinic based on the symptoms identified in their records.

### Hearing Health Theme

The ear, nose, and throat (ENT) clinic is interested in producing better structured data for patient records. Of particular interest is the ability to build custom phenotypes that are not easily captured in any medical ontology, such as SNOMED CT. For this project, CogStack annotations are being used to identify diagnoses and symptoms from the ENT free-text notes (letters and clinic notes). These extracted terms will in turn be used to build the phenotypes that the ENT clinic are interested in capturing.

### Clinical Coding

CogStack is working alongside the clinical coding team to build an interface that can help speed up the coding workflow. The interface is powered by UCLH’s MedCAT model that can identify clinical codes (International Classification of Diseases, 10th Revision [ICD10] codes) from a patient’s records (free-text notes, problem lists, etc). The interface will provide 2 important features. The first is the ability to automatically suggest clinical codes that should be assigned to the patient. These can be accepted or rejected by the coder, and this feedback can in turn be used to improve the software’s accuracy. The second feature is improved free-text searching across the patient’s records. The longer-term objective is that this interface could potentially replace the existing interface that coders are using and speed up the coding process.

### Identifying Clinical Intent in Free-Text Notes

Many patients often get “lost” in the system because a clinical order/appointment was not followed up. This happens for several reasons, such as the clinician not having undertaken the follow-up action (booking a scan, appointment, etc). In this project, CogStack is working with the Bariatrics clinic to train a machine learning model to predict a clinician’s intent to produce a follow-up action based on free-text notes. The system will scan through each clinical note and be able to see if the clinician has expressed an intent to produce some action, such as requesting an imaging procedure or discussing an item in a multidisciplinary team meeting. For many of these intents, one will be able to see if the intent was followed up, as many of them will have associated orders (imaging orders) on the hospital’s EHR system. The model will ultimately enable us to have a better understanding of where there are common gaps between intent and action, and ultimately improve patient care

### Atrial Fibrillation

Antithrombotics are blood thinning medications that are used to treat a range of cardiovascular diseases. Atrial fibrillation (AF) is one such disease and is the most common disturbance of heart rhythm and a common cause of stroke. In individuals who have AF, antithrombotics are used to lower stroke risk. However, around 1 in 5 of those with AF are not on the most effective type of antithrombotic or take no medication at all [25].

An NLP pipeline based on CogStack has been built to analyze 1.4 million hospital discharge summaries and automatically identify individuals with AF taking suboptimal medication. The pipeline is currently being tested at several other NHS Trusts and provides a framework for automated service evaluations and individual alerts for suboptimal medication.

## Discussion

In this paper, we have discussed UCLH’s deployment of the low-cost, open-source, text analytics information retrieval platform CogStack. We have discussed the need for such a platform, namely the issues of ingesting data from multiple systems, the heterogeneity in data sources, and, most importantly, text mining from the unstructured data. We have described how the platform has been adapted at UCLH and, in particular, have paid attention to the recent additions of the Apache NiFi module and the MedCAT modules.

We have described our deployment and how we have configured the tools provided by CogStack within our own hospital environment. The way in which we have configured the platform reflects the range of use cases that we are currently supporting and expect to support within the hospital. For example, our Apache NiFi data flows do not currently have a live data feed from the EHR system. This reflects the fact that all our use cases to date have been retrospective studies of EHR records or use cases where a live data feed is not required. Should we however require such a feed, UCLH has a live data warehouse, called EMAP [26], from which CogStack could read its records.

As demonstrated in the Results section, CogStack has previously supported and is currently successfully supporting a wide range of clinical use cases. Consequently, we feel that due to the low-cost requirements of both the platform and the NLP models available with the platform, CogStack can be deployed in most research-focused health care organizations. To assist other sites/individuals wishing to deploy the CogStack platform, the CogStack development team has recently launched a series of guides and an online forum [15,27,28].

## Abbreviations

AF: atrial fibrillation
API: application programming interface
DPIA: data protection impact assessment
EHR: electronic health record
ENT: ear, nose, and throat
ETL: extract, transform, and load
NHS: National Health Service
NLP: natural language processing
NPH: normal pressure hydrocephalus
SNOMED CT: Systematized Nomenclature of Medicine -- Clinical Terms
UCLH: University College London Hospitals
